# The Prevention of Tuberculosis

**Published:** 1892-01

**Authors:** Henry R. Hopkins

**Affiliations:** Buffalo, N. Y., Professor of Hygiene, Medical Department University of Buffalo; 433 Franklin Street


Buffalo Medical and Surgical Journal

VOL. XXXI. JANUARY, 1892. No. 6.

### Original Communications.

### THE PREVENTION OF TUBERCULOSIS.

BY HENRY R. HOPKINS, M. D., Buffalo, N. Y.,
Professor of Hygiene, Medical Department University of Buffalo.

"Millions for defense, but not one cent for tribute."—*Pinckney.*

IT MAY truthfully and pertinently be said that, as a people, we Americans are mightily absorbed in moneygetting and in money-spending display. Any new thought, no matter how intrinsically important it may be, which for a moment distracts or occupies the busy American from his favorite pastime of money-getting or display, finds it exceedingly difficult to get a hearing. In no department of economics is this peculiarity more distinctly marked than in the department of public health.

It would be easy and natural to suppose that the medical profession would be entirely exempt from any such pre-occupation as we have just referred to, and that any new discovery or any important statement of truth in such a preëminently practical direction as the prevention of disease, would find the medical profession not only eager to hear but quick to act. The study of state medicine, the practical work in the prevention of disease, only too clearly illustrates that the medical profession is human in its membership, and being human reflects quite too frequently the indifference of the community from which this medical profession springs and among which it labors. These reflections come to the writer's mind in the attempt to concentrate a few thoughts upon the most pertinent question in state medicine,— the prevention of tuberculosis.

Let me remind my readers that it is now nearly a quarter of a century since Villemin gave to the world the results of his interesting original experiments, in which he, in the most masterly manner, demonstrated the inoculability of tuberculosis. As the result of this original and preëminently important work, Villemin announced: First—As declared by Bayle and Lænnec, tuberculosis is a specific disease. Second—As demonstrated by myself, tuberculosis is an inoculable disease and must take its place among the virulent diseases, small-pox, scarlet fever and syphilis. Third—That tuberculosis arises by direct inoculation, by contagion, that its contagia are germs contained in the tuberculous matter or floating in the air. I would also remind my readers that in October, 1867, there appeared in the *London Lancet*, a publication by Dr. Wm. B. Budd, of England, which publication, rightly appreciated and loyally adopted, should have been a chart and compass for every board of health, municipal, state or national, as well as the guiding star for every individual member of the medical profession in the civilized world so far, at least, as the subject of tuberculosis is concerned. I give Dr. Budd's conclusions complete:

First: That tuberculosis is a true zymotic disease of specific nature in the same sense that typhoid fever, scarlet fever, typhus and syphilis are.

Second: That like these diseases tubercle never originates spontaneously, but is perpetuated solely by the law of contagious succession.

Third: That tuberculous matter itself is (or includes) the specific matter of the disease and constitutes the material by which phthisis is propagated from one person to another, and disseminated throughout society.

Fourth: That the deposits of this matter are, therefore, of the nature of an eruption and bear the same relation to the disease, phthisis, as the yellow matter (the stools) to typhoid fever.

Fifth: That by the destruction of this matter, on its issue from the body by means of proper chemicals or otherwise, seconded by good sanitary conditions, there is reason to hope that we may eventually, and possibly at no very distant time, rid ourselves entirely of this fatal scourge.

On April 10, 1882, Robert Koch published in Berlin his most important discovery of the existence and the pathogenesis of tubercle bacillus, since which time there has been neither confusion nor uncertainty as to the practical question of the infectiousness of tuberculosis, or as to the media by which such infection is carried on. The medical profession styles itself, and properly, a learned profession, and the American people considers itself one of the most enlightened of the civilized peoples of the world; yet, although nearly a decade has elapsed since the demonstration of the specific and infectious character of tuberculosis, I am not aware of the first movement being made on the part of any organized association of medical men, or on the part of any board of health, municipal or state, to put in operation the first practical measure towards the carrying into effect of the simple precautions announced nearly a quarter of a century ago.

To the writer it is hard to explain this indifference which looks amazingly like profound ignorance. That the question is eminently a practical one, will require but a few moments' scanning of undisputed statistics to demonstrate.

Our last census shows us that nearly one hundred thousand people die yearly from tuberculosis. It is a well-known fact that of all the diseases with which the medical profession have to contend, which ultimately result in loss of life, tuberculosis is one of the most chronic, and it is probably a safe estimate to assume that the period of incapacity for productive labor in each of the foregoing decedents was at least ten years. What does this annual loss mean in diminished productive labor? It simply means that every year there is a loss to this country by this disease alone of the labor of 1,000,000 of people for one year.

Let us now endeavor to put a pecuniary value upon this lost energy. Place the individuals earning at a low and safe figure — $200 per year. We find that we are paying a tax, levied and collected by a foreign power, of $200,000,000 per year, and, think of it, we used to be proud of the saying of our forefathers, "Millions for defense, but not one cent for tribute." In making this attempted estimate, no allowance is made for the loss from productive industry of those engaged in the nursing of the sick; nor for the sums expended for medicines, medical appliances, or for professional attention and other expenses incidental to the forlorn hope of bringing relief to the suffering. Will we not be entirely within bounds in estimating that these items will double our original sum, and that we will have an annual tribute levied upon us by tuberculosis at the sum of $400,000,000? Let us see if we cannot further illustrate the annual cost of tuberculosis by reference to the destruction of life and property by war. The Austria-Prussian war of 1866 cost in human life, 51,000 men, and $100,000,000. The Franco-German war of 1870-71 cost 311,000 men and $1,580,000,000. The war of the Rebellion from 1861-65 cost the north 280,000 men and $6,500,000,000. The annual death-rate of comsumptives each year will be seen to be greater by far than the death-rate during the war of the Rebellion, and the annual cost larger than the yearly expense of the armies and navies of the Empires of Russia, Great Britain and Germany. Students of sanitary science know very well that this is only one-third of the picture; that the death-rate and the expense-rate due to tuberculosis is more than doubled by the other zymotic preventable diseases.

It would seem that the time had fully come when the medical profession should arouse itself to the important practical question of "What shall be done to prevent this terrible waste of life and productive energy?" Is it not high time that the alarm should be sounded, and that the voice of alarm should never be still until the public torpor and public indifference be overcome and suitable measures be put into operation,—measures which are exceedingly simple and which in no degree rest upon hypothetical or theoretical foundations?

Should this question ever be seriously undertaken, it will at once appear that boards of health must be organized and raised to the highest possible point of efficiency; that such boards of health must be found in every municipality, in every state, and more important than any other, at the seat of the general government, and that the question cannot be considered with thorough efficiency until such boards of health are not only national but international. Our citizens willingly consent to be taxed for the maintenance of schools, for the training of soldiers and sailors to defend our homes against the destruction of war. Have we not an equal right and a greater reason to tax ourselves to maintain a National Board of Health, which board shall have for its object the defense of the otherwise defenseless, against the incursion of tuberculosis and other preventable diseases?

433 FRANKLIN STREET.

---

DR. J. B. MATTISON (*Northwestern Lancet*) states that cocaine has proved fatal in smaller dose than morphia. From the analysis of a large number of cases he concludes that the fatal dose of cocaine is uncertain, dangerous, and even deadly results following doses usually deemed safe. The age or condition of the patient appears to have no constant relation to the development of poisonous effects, but the danger is decidedly greatest when the drug is given under the skin and in persons with renal or cardiac weakness.

